# Enhanced genomic stability of new miRNA-regulated oncolytic coxsackievirus B3

**DOI:** 10.1016/j.omto.2022.10.003

**Published:** 2022-10-08

**Authors:** Huitao Liu, Amirhossein Bahreyni, Yasir Mohamud, Yuan Chao Xue, William W.G. Jia, Honglin Luo

**Affiliations:** 1 Centre for Heart Lung Innovation, St Paul’s Hospital, Vancouver, BC V6Z 1Y6, Canada; 2 Department of Experimental Medicine, University of British Columbia, Vancouver, BC V6Z 1Y6, Canada; 3 Department of Pathology and Laboratory of Medicine, University of British Columbia, Vancouver, BC V6Z 1Y6, Canada; 4 Virogin Biotech Ltd, Vancouver, BC V6S 2L9, Canada

**Keywords:** oncolytic virus, coxsackievirus B3, miRNA, genomic stability, oncolytic activity, cytotoxicity, lung cancer

## Abstract

Genetic modification of coxsackievirus B3 (CVB3) by inserting target sequences (TS) of tumor-suppressive and/or organ-selective microRNAs (miRs) into viral genome can efficiently eliminate viral pathogenesis without significant impacts on its oncolytic activity. Nonetheless, reversion mutants (loss of miR-TS inserts) were identified as early as day 35 post-injection in ∼40% immunodeficient mice. To improve the stability, here we re-engineered CVB3 by (1) replacing the same length of viral genome at the non-coding region with TS of cardiac-selective miR-1/miR-133 and pancreas-enriched miR-216/miR-375 or (2) inserting the above miR-TS into the coding region (i.e., P1 region) of viral genome. Serial passaging of these newly established miR-CVB3s in cultured cells for 20 rounds demonstrated significantly improved stability compared with the first-generation miR-CVB3 with 5′UTR insertion of miR-TS. The safety and stability of these new miR-CVB3s was verified in immunocompetent mice. Moreover, we showed that these new viruses retained the ability to suppress lung tumor growth in a xenograft mouse model. Finally, we observed that miR-CVB3 with insertion in P1 region was more stable than miR-CVB3 with preserved length of the 5′UTR, whereas the latter displayed significantly higher oncolytic activity. Overall, we presented here valid strategies to enhance the genomic stability of miR-CVB3 for virotherapy.

## Graphical abstract

**Figure:**
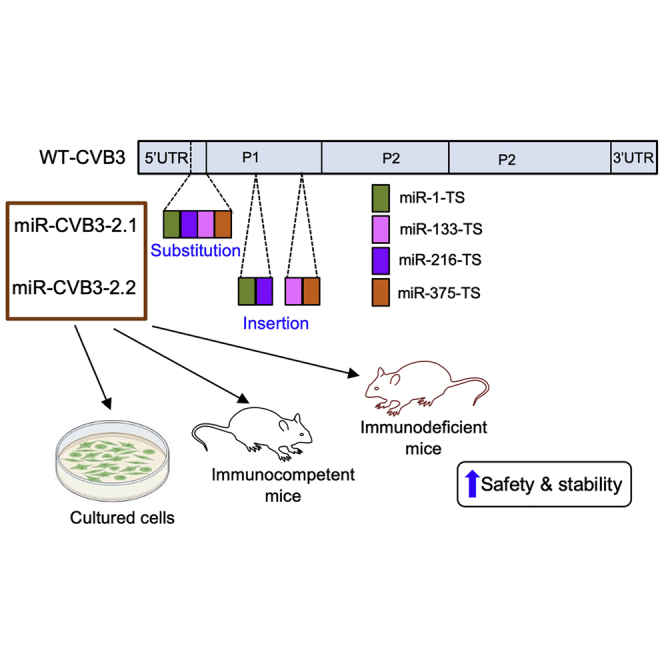

## Teaser

Liu et al. developed two new versions of miRNA-modified coxsackievirus B3, which exhibit significantly improved stability of the inserted miRNA target sequence both *in vitro* and in immunocompetent mice, while retaining the oncolytic potency against small cell lung cancer in a xenograft mouse model.

## Introduction

Coxsackievirus B3 (CVB3) has emerged as an attractive agent for cancer therapy due to its strong oncolytic ability against multiple types of tumors, including lung cancer,^1^^,^^2^ breast cancer,^3^ and colorectal cancer.^4^ Nonetheless, virus-induced pathogenesis, including myocarditis and pancreatitis, confines its application as an oncolytic virotherapy tool. Among different approaches to decrease viral toxicities,^5^ genetic modification through inserting target sequences (TS) of tumor-suppressive microRNAs (miRs), such as miR-143/miR-145 ^6^ and miR-34,^7^ or organ-selective miRs, such as cardiac-enriched miR-1 and pancreatic-specific miR-216/miR-375,^3^^,^^8^^,^^9^ into the non-coding (5′UTR or 3′UTR) or coding regions of CVB3 genome, has proven to be extremely effective in reducing organ toxicity and enhancing its specificity toward cancer cells.

CVB3 is a positive, single-stranded RNA virus in the family of *picornaviridae* with a small genome size of around 7.4 kb. Like other picornaviruses,^10^ CVB3 has limited packaging capacities and is inclined to eliminate exogenous genomic insertion during viral replication, resulting in reversion mutations.^5^ We have previously reported the loss of miR-145/143-TS, which was inserted into the 5′UTR of CVB3 genome, in approximately 40% of mice after extended period of treatment (i.e., >35 days), suggesting genomic instability of this version of miR-CVB3.^6^ We speculated that two reasons may account for this phenomenon. First, the length of CVB3 5′UTR is evolutionarily optimal, so addition of exogenous miR-TS in this region leads to reduced genomic stability of miR-CVB3. Second, as 5′UTR does not encode viral proteins, insertion within 5′UTR is less stable than that in coding region during viral genome replication.

The goal of the current study is to re-engineer CVB3 with miR-TS to enhance the stability of miRNA-modified CVB3. To achieve this goal, we used two strategies to construct recombinant CVB3s: (1) substituting the same length of 5′UTR at the ribosomal scanning region between internal ribosome entry site (IRES) and start codon with TS of cardiac-selective miR-1/miR-133 and TS of pancreas-enriched miR-216/miR-375 to preserve the genome length of 5′UTR and prevent possible disruption to neighboring RNA structure and (2) inserting miR-TS into the P1 coding region of viral genome. We assessed the stability, safety, and anti-tumor activity of these second-generation miR-CVB3s. *In vitro* and animal studies demonstrated that the new miR-CVB3s displayed significantly improved safety and genetic stability profile while maintaining the oncolytic activity targeting small cell lung cancer (SCLC).

## Results

### Construction of the second-generation miR-CVB3s

miR-1/133 and miR-216/375 have been shown to be highly expressed in the heart and pancreas, respectively.^11^^,^^12^ These miRs are also known to be frequently downregulated in cancerous cells, and they function as tumor suppressors by negatively regulating the expression of many oncogenes.^13^ In this study, we constructed the second-generation miR-CVB3s using these organ-selective miR-TS.

**Figure 1:**
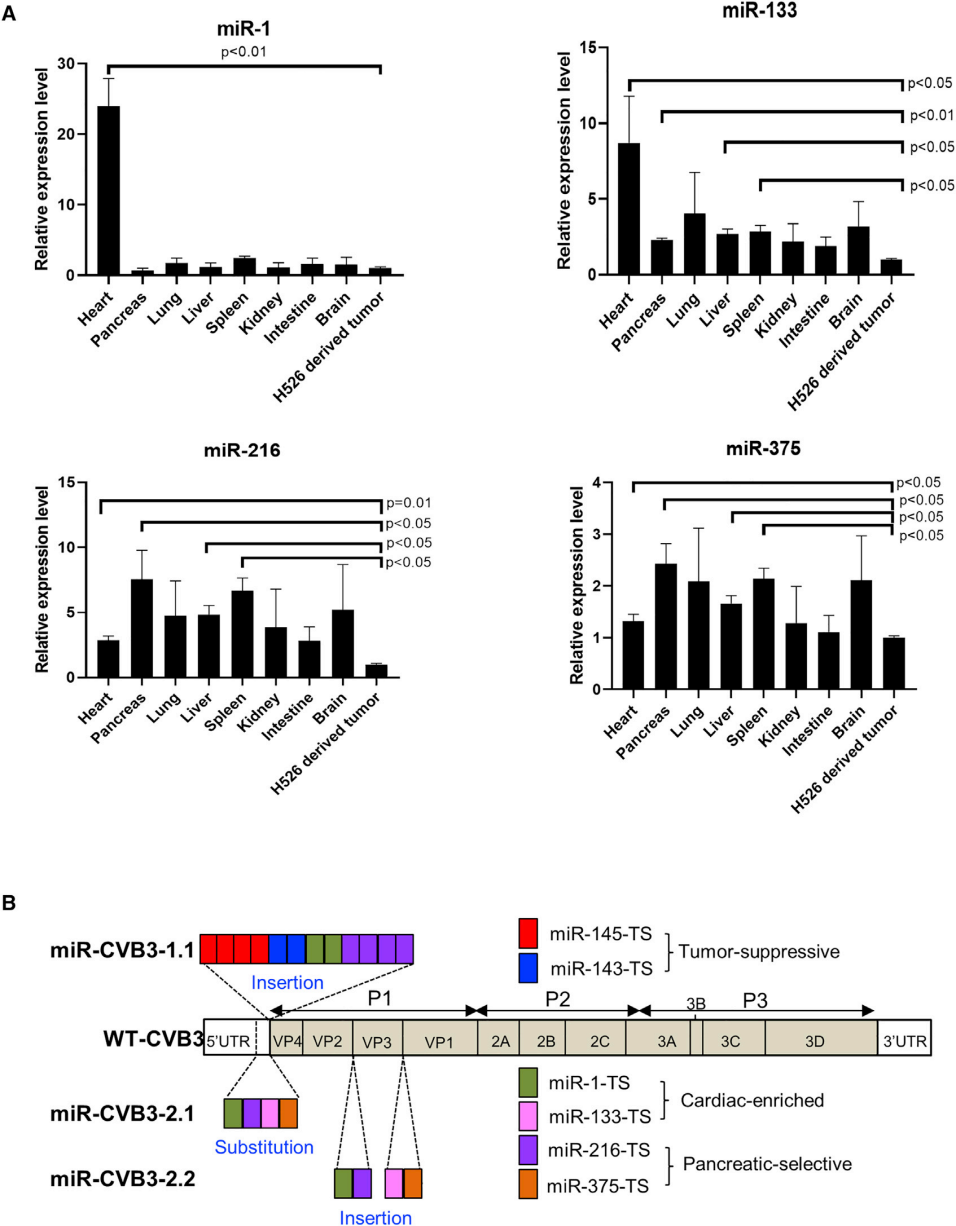
Construction of the second-generation miR-CVB3s (A) Relative expression of miR-1, miR-133, miR-216, and miR-375 in various NOS-SCID mouse tissues and in H526-derived xenograft tumors was measured by qRT-PCR and presented as mean ± SD (n = 3). (B) New miRNA-modified CVB3s (miR-CVB3s), namely miR-CVB3-2.1 and miR-CVB3-2.2, were generated by substituting viral genomic RNA within 5′UTR with target sequences (TS) of organ-specific miRs (miR-CVB3-2.1) or inserting these organ-specific miR-TS into P1 region of viral genome (miR-CVB3-2.2) as indicated. miR-CVB3-1.1 was previously generated by inserting tumor-suppressive and organ-selective miR-TS into the 5′UTR of CVB3 as indicated and used as a control.

To verify organ-selective expression, we examined the levels of miR-1, miR-133, miR-216, and miR-375 in various mouse organs and H536-derived tumor tissues by qRT-PCR. Consistent with previous reports,^11^ we demonstrated that miR-1 and miR-133 were significantly abundant in the heart compared with other organs and tumor tissues (Figure 1A). We also showed that the expression levels of miR-216 and miR-375 were significantly higher in the pancreas than in the tumor tissues (Figure 1A).

To generate the second-generation miR-CVB3s (Figure 1B), we constructed the miR-CVB3-2.1 by replacing the last 88 nucleotides of the CVB3 5′UTR (i.e., ribosomal scanning region after domain VI and before start codon, which is weaker structured and whose sequence is not essential for picornaviral replication^14^, ^15^, ^16^) with TS of miR-1, miR-133, miR-216, and miR-375 (one copy of each). The miR-CVB3-2.2 was formulated through inserting TS of the above miRs into the P1 region (i.e., 1× miR-1-TS and 1× miR-216-TS between VP2 and VP3, and 1× miR-133-TS plus 1× miR-375-TS between VP3 and VP1). The miR-CVB3-1.1 was generated previously,^17^ by inserting 4× miR-145-TS, 2× miR-143-TS, 2× miR-1-TS, and 4× miR-216-TS into the 3′ terminus of CVB3 5′UTR (Figure 1B).

### Assessment of genomic stability of newly generated miR-CVB3s *in vitro*

**Figure 2:**
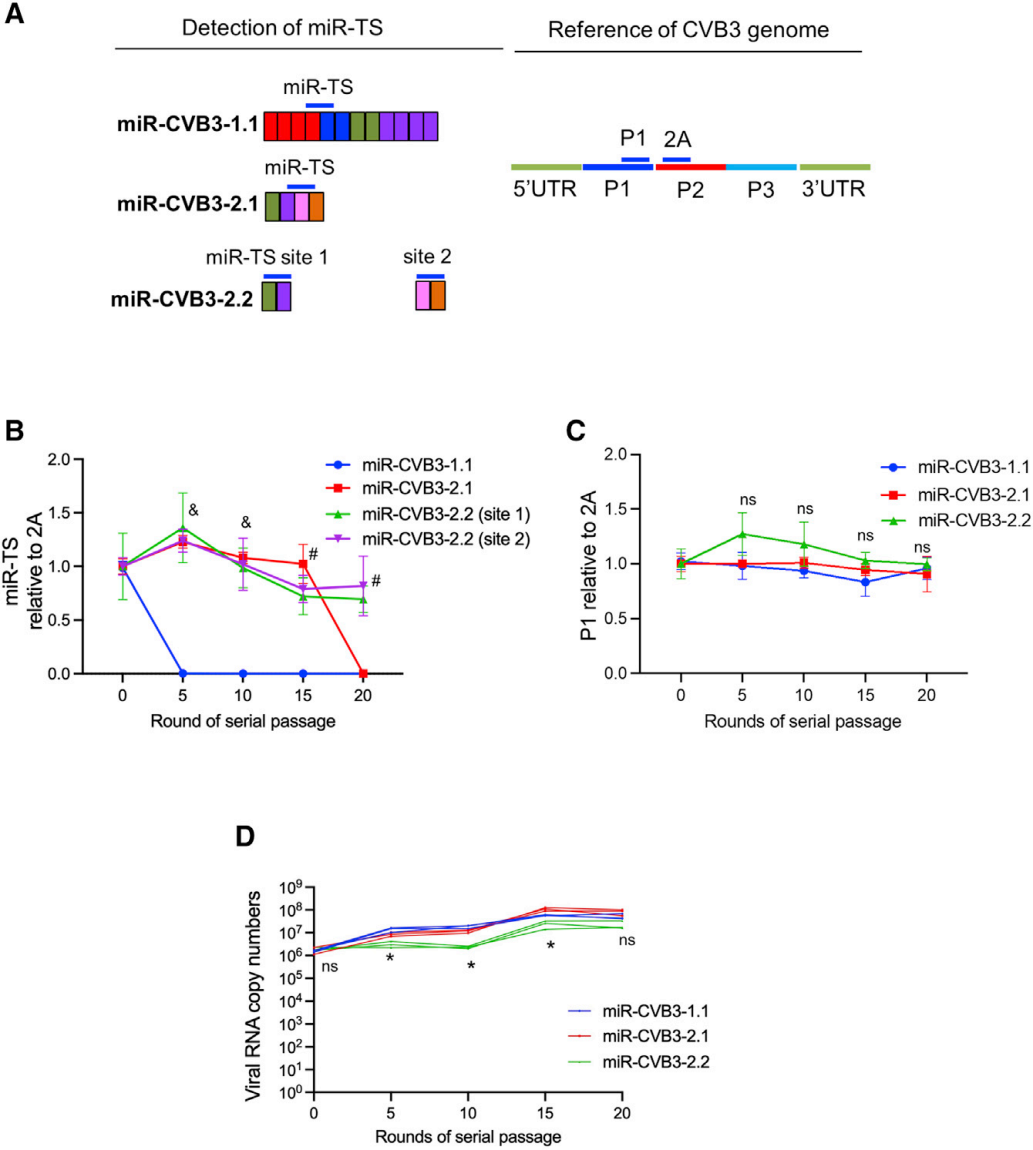
Measurement of genomic stability of newly generated miR-CVB3s *in vitro* (A) Illustration of the primer binding sites (blue lines) on TS of indicated miR-CVB3s. Primers binding to P1 region or 2A within P2 region were used as references (internal controls). Note that two primer pairs (denoted as “site 1” and “site 2”) were designed to assess the integrity of miR-CVB3-2.2 by monitoring the existence of two separate miR-TS inserts. (B, C) miR-CVB3s were serially passaged in HL-1 cardiomyocytes for up to 20 passages. Cell supernatants were harvested every five passages for viral genome quantification by qRT-PCR using primers as described above targeting miR-TS (B) or P1 region (C) relative to the copy numbers of 2A (mean ± SD, n = 3). (D) Absolute quantification of viral genome copy numbers in the supernatants was determined using primers targeting 2A (mean ± SD, n = 3 for each group). ∗, p < 0.05; #, p < 0.01; &, p < 0.005 compared with miR-CVB3-1.1. ns, not significant.

Due to the increased selective pressure, HL-1 mouse cardiomyocytes, which express miR-1 and miR-133,^18^ were selected to evaluate the stability of miR-CVB3s. Briefly, different miR-CVB3s were serially passaged in HL-1 cells for up to 20 passages. Supernatants were harvested every five passages for viral genome quantification by qRT-PCR with primers targeting specific miR-TS or P1 region referenced to viral 2A (Figure 2A).

Figure 2Bshowed quantification of miR-TS of different miR-CVB3. The value of “1.0” indicates intact primer binding sites on miR-TS, whereas “0” denotes reversion mutation or loss of miR-TS. The number between “1.0” and “0” suggests reduced viral genome stability. As shown in Figure 2B, after five-round passaging, the value of miR-CVB3-1.1 reached “0,” indicating complete loss or instability of miR-TS. In contrast, miR-CVB3-2.1 was stable until passage 15 and miR-CVB3-2.2 until at least passage 20, suggesting great improvement of the stability of new-generation of miR-CVB3s. As the miR-TS in miR-CVB3-2.2 were inserted into two sites in P1 region, the stability of the two inserts was examined separately using two primer pairs. As comparison, relative quantification of P1 to 2A as an internal control remained around 1.0 across the 20-round passaging (Figure 2C). The absolute quantification of viral genome copies presented in Figure 2D revealed that all viruses were able to replicate efficiently across the 20-round passaging, excluding the possibility that reduction in copy number of miR-TS of miR-CVB-1.1 and miR-CVB3-2.1 shown in Figure 2B was a result of halted viral replication.

### Evaluation of lytic and replicative ability of new miR-CVB3s *in vitro*

**Figure 3:**
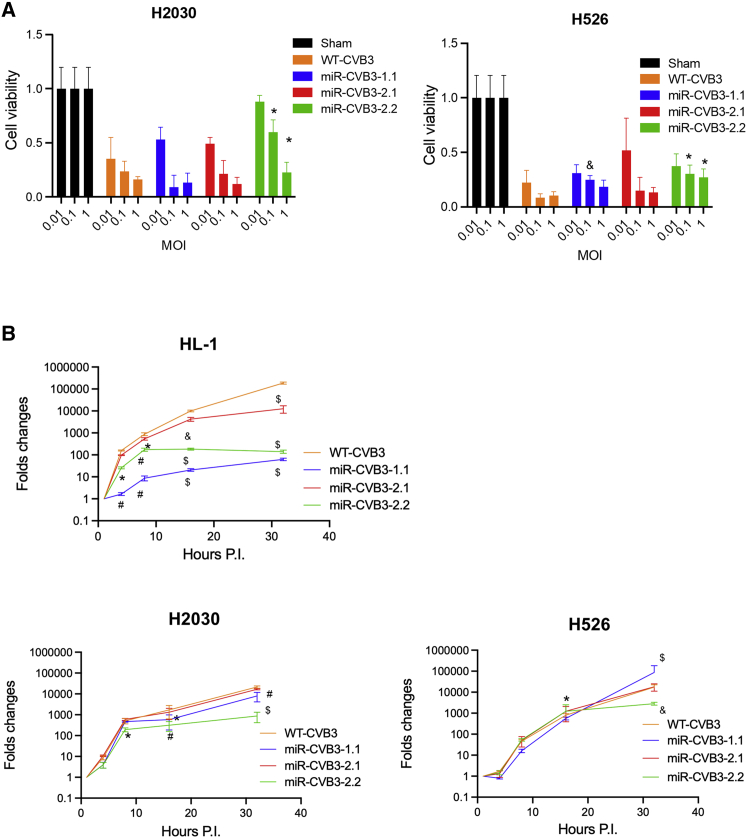
Evaluation of lytic and replicative ability of new miR-CVB3s *in vitro* (A) *KRAS*^*mut*^ lung adenocarcinoma H2030 and *TP53*^*mut*^*/RB1*^*mut*^ SCLC H526 cell lines were sham-infected or infected with indicated WT- or miR-CVB3s at MOI = 0.01, 0.1, 1 for 72 h. Cell viability was assessed by alamarBlue assay (mean ± SD, n = 3). (B) HL-1 cardiomyocytes, H2030, and H526 lung cancer cells were inoculated with WT- or miR-CVB3s at MOI = 0.01 for indicated hours. Cell lysates were harvested for RNA extraction and quantification of viral genome by qRT-PCR using 2A primer pairs. The viral copy number at day 1 is set arbitrarily to 1 and fold changes are presented throughout the time course (mean ± SD, n = 3). Unpaired Student’s t test was conducted. ∗, p < 0.05; #, p < 0.01; &, p < 0.005; $, p < 0.001 compared with WT-CVB3. PI, post-infection.

After confirming the stability, we next sought to examine the anti-tumor lytic activity of new miR-CVB3s. Human *KRAS*^*mut*^ lung adenocarcinoma H2030 and *TP53*^*mut*^*/RB1*^*mut*^ SCLC H526 cells were sham-infected or incubated with wild-type (WT)-CVB3, miR-CVB3-1.1, miR-CVB3-2.1, or miR-CVB3-2.2 at a multiplicity of infection (MOI) of 0.01, 0.1, and 1 for 72 h. Cell viability assay demonstrated the effectiveness of these new miR-CVB3s in lysing tumor cells, especially at the MOI of 1, albeit at a slightly reduced level relative to WT-CVB3 (Figure 3A).

To further determine the replication kinetics of miR-CVB3s, we conducted qRT-PCR to measure growth curve of viral genome over a 32-h experimental period. HL-1 cardiomyocytes, H2030, and H526 cells were infected with WT-CVB3 or different miR-CVB3s at an MOI of 0.01 for various times, followed by qRT-PCR quantification of viral genome using primers targeting 2A. We found that viral RNA levels were substantially lower in HL-1 cells inoculated with various miR-CVB3s compared with WT-CVB3 (Figure 3B). It was noted that, between the two new miR-CVB3s, replication of miR-CVB3-2.2 (similar observation was made for miR-CVB3-1.1) in HL-1 cells was significantly less efficient than that of miR-CVB3-2.1, even though they harbor the same miR-TS (Figure 3B). Furthermore, we observed that RNA copy numbers of miR-CVB3-2.1 and miR-CVB3-2.2 were comparable or modestly reduced in H2030 and H526 cells compared with WT-CVB3 (Figure 3B). Together, these results suggest that the second-generation miR-CVB3s retain the oncolytic and replication activity in lung cancer cells, whereas their ability to replicate in cardiomyocytes was drastically attenuated compared with WT-CVB3.

### Safety measurement of new miR-CVB3s in immunocompetent mice

We next examined the safety profile of new miR-CVB3s in immunocompetent mice. A/J mice, a mouse strain known to be susceptible to CVB3 infection,^19^ were inoculated intraperitoneally with PBS, WT-, or miR-CVB3s once at a dose of 1 × 10^6^ plaque-forming units (PFU) for up to 14 days (experimental endpoint). A pilot study showed that body weight of mice treated with miR-CVB3-1.1 declined drastically after day 4 post-infection similar to WT-CVB3 (not shown), suggesting a significant toxicity in this strain of mice. We therefore decided to focus on the second-generation miR-CVB3s for detail characterization of their safety profile.

**Figure 4:**
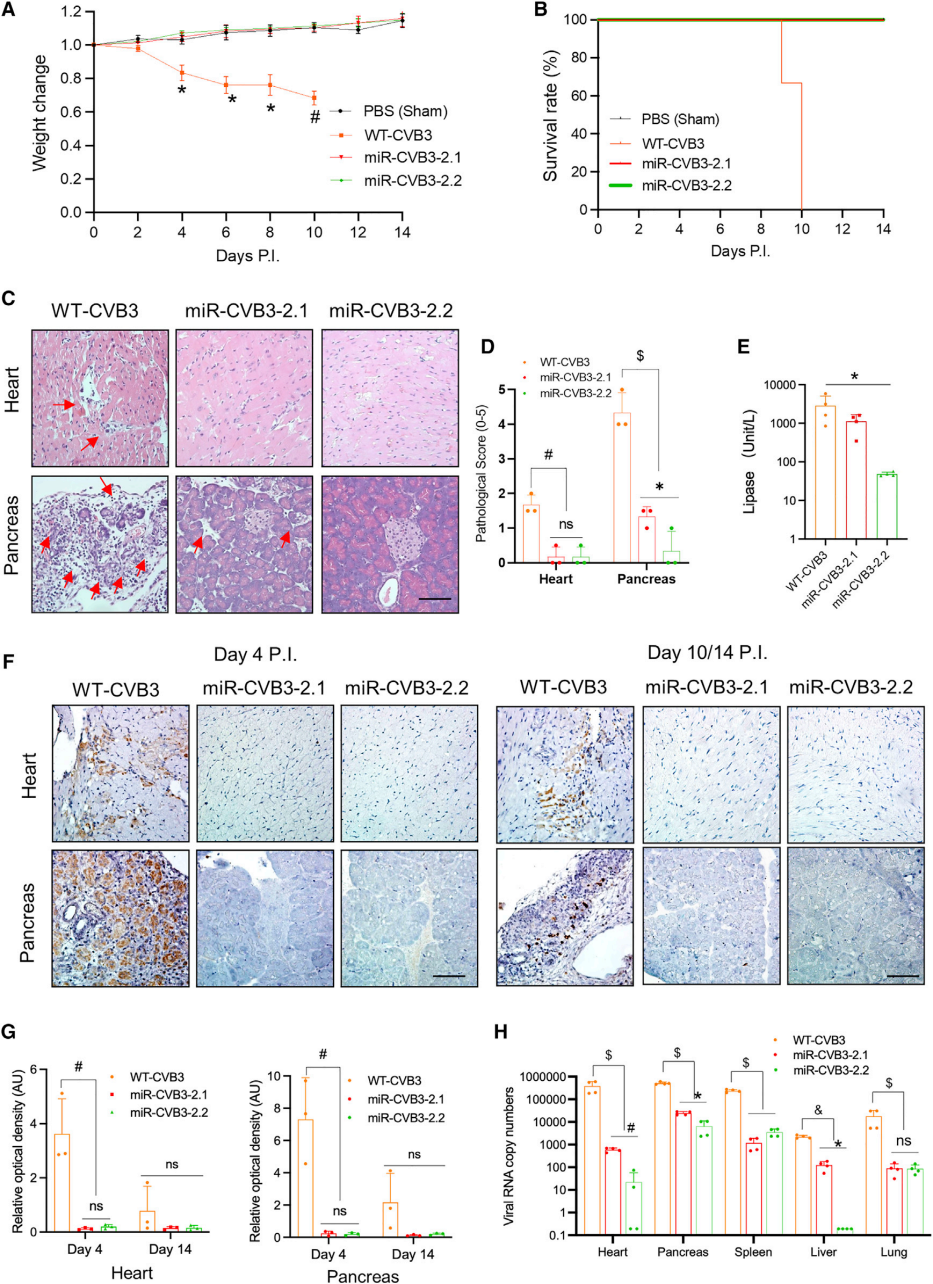
Safety evaluation of new miR-CVB3s in immunocompetent mice A/J mice were inoculated intraperitoneally with PBS (sham infection), WT- or miR-CVB3s at a dose of 1 × 10^6^ PFU for up to 14 days. (A) Body weight was measured every other day (mean ± SD, n = 3 for each group) and normalized to that on day 0, which was arbitrarily set a value of 1.0. (B) Kaplan-Meier survival rate (n = 3 for each group) was recorded and plotted daily until experimental endpoint (day 14 following treatment). p < 0.05 comparing survivals in WT-CVB3 group with those in PBS, miR-CVB3-2.1, or miR-CVB3-2.2 group by log rank test. (C, D) H&E staining was performed on the heart and pancreas collected at day 4 post-treatment from a different cohort of mice (C), and pathological scores (mean ± SD, n = 3 mice) were evaluated based on the H&E staining (D). Arrows indicate tissue damage and inflammatory infiltration. (E) Blood was collected at day 4 post-infection (mean ± SD, n = 4 mice) for biochemical analysis of serum lipase levels using Advia 1800 autoanalyzer. (F, G) Immunostaining was conducted to assess viral protein VP1 expression in mouse heart and pancreas harvested on day 4 and 10/14 (WT-CVB3 and miR-CVB3, respectively) post-infection (F), and relative optical density of VP1 (mean ± SD, n = 3 mice) based on immunostaining images was quantified (G). (H) qRT-PCR was performed to measure viral RNA levels in various organs harvested from mice treated with miR-CVB3-2.1, miR-CVB3-2.2, or WT-CVB3 at day 4 post-infection (mean ± SD, n = 3 mice). ∗, p < 0.05; #, p < 0.01; &, p < 0.005; $, p < 0.001 compared with WT-CVB3. ns, not significant. PI, post-infection. Scale bar represents 100 μM.

Figures 4A and 4B showed that mice injected with miR-CVB3-2.1 or miR-CVB3-2.2 exhibited a similar body weight increase as sham-infected mice, and all mice survived the 2-week monitoring period. In contrast, mice treated with WT-CVB3 started to lose weight at day 4, and all died or euthanized due to severe viral virulence at day 9–10 after treatment.

We further carried out hematoxylin and eosin (H&E) staining to assess possible tissue toxicities (Figure 4C). Pathological quantitation of the H&E staining revealed tissue damage and inflammatory infiltration in the heart and pancreas of mice, in particular the latter, at day 4 following WT-CVB3 treatment. However, cardiac and pancreatic pathogenesis was markedly reduced to a significantly lower level in mice treated with either miR-CVB3-2.1 or miR-CVB3-2.2 (Figure 4D). Biochemical analysis of serum lipase levels confirmed attenuated pancreatic toxicity particularly with miR-CVB3-2.2 treatment (Figure 4E). Likewise, immunostaining showed that viral protein VP1 expression was greatly decreased in the heart and pancreas of mice treated with miR-CVB3-2.1 or miR-CVB3-2.2 in comparison with WT-CVB3-treated mice (Figures 4F and 4G). As expected, the level of VP1 was significantly lower on day 10 (for WT-CVB3) or 14 (for miR-CVB3) than that on day 4 as a result of viral clearance (Figures 4F and 4G). Furthermore, qRT-PCR analysis validated the drastic reduction of viral genomic levels in various organs of mice treated with miR-CVB3-2.1 or miR-CVB3-2.2 compared with WT-CVB3-treated mice at day 4 post-infection (Figure 4H). Finally, sequence analysis of viral genome after 14-day infection revealed no reversion mutations and that the majority of the miR-TS remained functional, although several single mutations, particularly for miR-CVB3-2.1, were observed (Figure S1).

Collective, our results indicate that the new miR-CVB3s have an improved safety profile when used in immunocompetent mice at least for the time frame examined in this study.

### Evaluation of anti-tumor efficacy and safety of new miR-CVB3s in a xenograft mouse model

**Figure 5:**
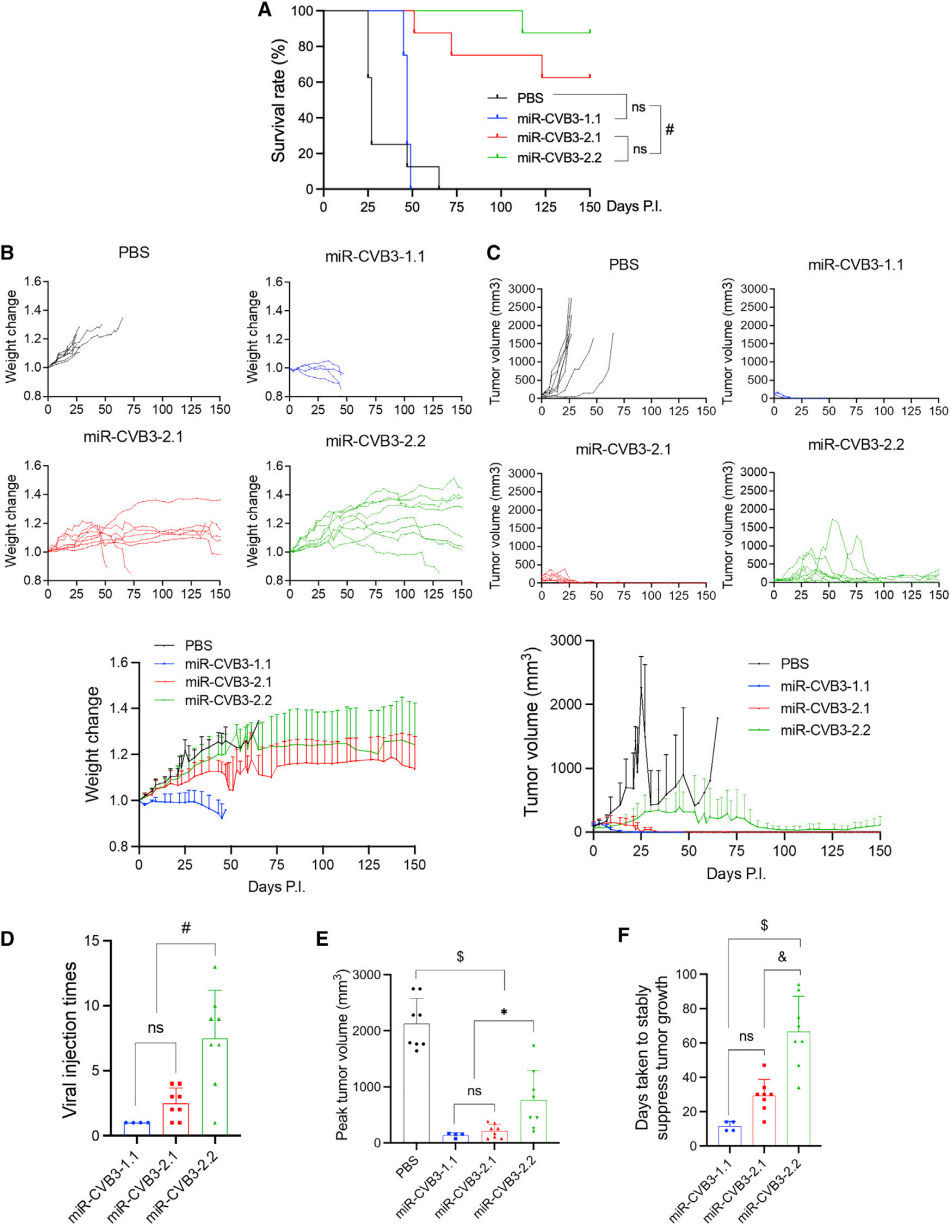
Assessment of safety and oncolytic efficacy of new miR-CVB3s in a xenograft SCLC mouse model SCID mice bearing H526-derived xenograft tumors were treated intraperitoneally once a week with PBS (n = 8), miR-CVB3-1.1 (n = 4), miR-CVB3-2.1 (n = 8), or miR-CVB3-2.2 (n = 8) at a dose of 1 × 10^6^ PFU. (A) Kaplan-Meier survival rate was monitored until experimental endpoint (day 150 post-treatment). (B) Body weight was measured twice every week and normalized to those on day 0 (mean ± SD). (C) Tumor volume was measured twice weekly until experimental endpoint (mean ± SD). (D) Average number of viral injections until tumor growth was firmly suppressed. (E) Peak tumor volumes in each group. (F) Days taken to stably suppress tumor growth for 2 weeks. ∗, p < 0.05; #, p < 0.01; &, p < 0.005; $, p < 0.001. ns, not significant. PI, post-infection.

We then evaluated the safety and anti-tumor effectiveness of the new miR-CVB3s in an SCLC xenograft mouse model. Immunocompromised non-obese diabetic-severe combined immune deficiency (NOD-SCID) mice bearing H526-derived SCLC xenografts were injected intraperitoneally with PBS, miR-CVB3-1.1, miR-CVB3-2.1, or miR-CVB3-2.2 at a dose of 1 × 10^6^ PFU weekly until the tumor growth was stably repressed (<100 mm^3^ for 2 weeks). Kaplan-Meier analysis revealed that the survival rates for mice treated with miR-CVB3-2.1 or miR-CVB3-2.2 at day 150 (experimental endpoint) were ∼62.5% and ∼87.5% respectively, while mice inoculated with miR-CVB3-1.1 all died or were euthanized because of severe viral toxicities prior to day 48 following treatment (Figure 5A). In PBS-treated group, mice were euthanized over the time course due to surpassed tumor size per animal care guideline. Figure 5B presented the changes in body weight (from individual mice or average) in each treatment group. Mice that underwent significant loss in body weight were euthanized.

The ability of miR-CVB3s to suppress xenograft tumors was assessed by tumor growth curve, which revealed a marked reduction in tumor volume upon treatment with different miR-CVB3s compared with PBS control (Figure 5C). We further analyzed viral injection times needed for steady and significant tumor suppression. Figure 5D showed that the average numbers of viral injection to achieve tumor suppression were comparable between miR-CVB3-1.1 and miR-CVB3-2.1, while significantly higher for miR-CVB3-2.2 group. The peak tumor volumes and days taken to repress the tumor growth were also examined. We found that the anti-tumor ability of miR-CVB3-1.1 and miR-CVB3-2.1 was significantly more profound than miR-CVB3-2.2 (Figures 5E and 5F).

Lastly, we performed miR-CVB3 sequencing to determine whether the mice that were euthanized or that died early prior to the experimental endpoint of day 150 is due to genome instability. We found almost complete loss of inserted miR-TS in miR-CVB3-1.1 at day 47 post-infection (Figure S2). Notably, miR-CVB3-2.1 and miR-CVB3-2.2 also displayed partial loss of the insertion, but at much later time-points (i.e., day 72 for miR-CVB3-2.1 and day 112 for miR-CVB3-2.2) compared with miR-CVB3-1.1 (Figure S2).

Taken together, our results indicate that both miR-CVB3-2.1 and miR-CVB3-2.2 are safe in mice and retain oncolytic capability with the former displaying a greater anti-tumor activity than the latter.

## Discussion

Various miRNA-regulated CVB3s have been generated by inserting miR-TS into the 5′UTR, 3′UTR, or coding regions of CVB3 genome.^3^^,^^6^, ^7^, ^8^, ^9^ However, their genomic stability was either unsatisfactory or uncharacterized. In this study, we formulated two miRNA-modified CVB3s, named miR-CVB3-2.1 and miR-CVB3-2.2, to test our hypothesis that incorporating miR-TS to the 5′UTR of CVB3 via substitution to preserve genome length of 5′UTR or inserting miR-TS into P1 region that encodes viral structural proteins enhances genetic stability of miR-CVB3s, respectively.

As indicated earlier, we have previously observed that miR-CVB3 (the first version of miRNA-modified CVB3), constructed by inserting four copies of miR-145-TS and two copies of miR-143-TS with a total length of 163 bp into the 5′UTR of CVB3, is genetic unstable after prolonged application to immunocompromised mice.^6^ In the present study, we assessed the genome stability of miR-CVB3-2.1 and miR-CVB3-2.2 in comparison with miR-CVB3-1.1. The miR-CVB3-1.1 was recently generated to improve the safety of the original miR-CVB3 in immunocompetent mice by inserting two copies of cardiac-specific miR-1-TS and four copies of pancreatic-specific miR-216-TS into the first version miR-CVB3, and its stability was not previously evaluated.^17^

Since 3′UTR has limited length for replacement with the miR-TS inserts, the current study utilized the 5′UTR for viral reconstruction. miR-CVB3-2.1 was generated by substituting 88 nucleotides between domain VII and start codon within the 5′UTR with a single copy of each TS of cardiac-enriched miR-1/miR-133 and pancreatic-selective miR-216/miR-375. miR-CVB3-2.2 was created by inserting the same TS into two sites of the P1 coding region. The selection of two different miRNAs highly expressed in the same organ is based on the consideration of safety redundancy in case one miR-TS loses its function. Here we demonstrated that compared with miR-CVB3-1.1, the miR-TS insertion was lost after five-round passaging, both miR-CVB3-2.1 and miR-CVB3-2.2 are stable, and the miRNA target sites can be detected even after 15-round passaging in cultured cells. Significantly improved safety of miR-CVB3-2.1 (62.5% survival on day 150, the experimental endpoint) and miR-CVB3-2.2 (87.5% survival on day 150) compared with miR-CVB3-1.1 (0% on day 48, the humane endpoint) was also validated in an SCLC xenograft mouse model.

Fechner group has previously described the formulation of a miR-CVB3 by inserting miR-375-TS into the polyprotein coding region of CVB3 genome.^9^ However, genomic stability of this recombinant CVB3 was not investigated. In this study, we provided the first evidence that insertion of miR-TS to the coding region of CVB3 genome significantly improves the stability of miR-CVB3. Elsedawy et al.^20^ at the Mayo Clinic College of Medicine recently reported that replacing a region of 5′UTR between IRES and start codon of coxsackievirus A21 with TS of muscle-specific miR-133/miR-206 increases the stability of miRNA target elements *in vitro* and *in vivo.*^20^ Using a similar approach, in this study we demonstrated that substitution of an equal length of 5′UTR with organ-specific miRNA TS augments genomic stability of miR-CVB3, verifying the effectiveness of this strategy at least for coxsackieviruses.

qRT-PCR instead of sequencing was used to monitor the presence or absence of miR-TS in miR-CVB3-infected mouse cardiomyocytes for convenience and simplicity. Although PCR can only detect the integrity of a partial region of miR-TS, it offers general information about whether the inserts of miR-TS are disrupted or lost. Combining with the sequencing data conducted on viral RNA extracted from mouse tissues, our results provide strong evidence that miR-CVB3-2.1 and miR-CVB3-2.2 are more stable than miR-CVB3-1.1.

While miR-CVB3-2.1 and miR-CVB3-2.2 contain the same miR-TS, viral growth kinetics and oncolytic activity appear very distinct. Insertion of miR-TS into the P1 coding region of viral genome produces a more attenuated but genomically more stable virus than 5′UTR substitution, similar to a previous report where the addition of miR-TS in viral structural protein coding region confers weaker viral fitness than miR-TS insertion into the 3′UTR.^9^ In addition to the difference in position of viral modification site, genomic size (miR-CVB-2.2 has a longer genomic length than miR-CVB3-2.1 that preserves the original length) may be another factor contributing to attenuation of miR-CVB3-2.2. Interestingly, miR-CVB3-1.1, which has the largest miR-TS insertion among the three miR-CVB3s, displays higher replication capacity than miR-CVB3-2.2 in tumor cells, suggesting that viral genome size may not be the sole factor in replication capacity. Future investigations are needed to address the precise mechanism of the greater growth defects of miR-CVB3-2.2 compared with miR-CVB3-2.1.

Pancreas has been regarded as an organ needed for the spread of CVB3 from the peritoneal cavity to other organs upon intraperitoneal injection.^21^ Our current and previous studies revealed that although tissue toxicity and viral RNA level were drastically reduced with miR-CVB3s, viral genome remained detectable in the pancreas.^6^^,^^17^ In addition, massive viral replication was observed in tumor tissues.^17^ Thus, we suspect that the presence of a moderate level of viral genome in the pancreas is sufficient to induce systemic infection.

In conclusion, we developed two new miR-CVB3s, which are genetically stable and preserve the oncolytic activity of the parental viruses against lung cancer.

## Materials and methods

### Cell lines

The H2030 lung adenocarcinoma epithelial cell line (#CRL-5914) and H526 SCLC epithelial cell line (#CRL-5811) were obtained from the American Type Culture Collection and cultured in Roswell Park Memorial Institute (RPMI) 1640 medium (#11875093, Thermo Fisher Scientific) supplemented with 10% FBS and 1% penicillin-streptomycin solution. The HL-1 mouse cardiomyocytes obtained from Sigma-Aldrich (#SCC065) were cultured in Claycomb medium (#51800C, Sigma-Aldrich) as previously described.^22^ Cell lines used in the current study were confirmed to be mycoplasma-free using a mycoplasma PCR Detection Kit (#G238, ABM).

### Generation of miRNA-CVB3 constructs

To generate miR-CVB3 construct, a linearized CVB3 plasmid (Kandolf strain) was first amplified by a primer pair around the insertion site. The miR-CVB3 was then assembled with an oligo pair including the miR-TS and necessary overlapping sequence using the NEBuilder HiFi DNA Assembly Master Mix kit (E2621, New England Biolabs) following the manufacturer’s protocol. To preserve the length of 5′UTR, miR-CVB3-2.1 was constructed by substituting 88-nucleotide between IRES and start codon at the 5′UTR with TS of miR-1, miR-133, miR-216, miR-375 (one copy of each without spacer in between) first using a primer pair (forward: 5′- ATACAGCAAAATGGGAGCTCA-3′, reverse: 5′- CACCGGATGGCCAATCCAAT-3′) and then an oligo pair (fragment 1: 5′-ATT GGA TTG GCC ATC CGG TGA TAC ATA CTT CTT TAC ATT CCA TCA CAG TTG CCA GCT GAG ATT ATA GCT GGT TG-3′, fragment 2: 5′- TGA GCT CCC ATT TTG CTG TAT TTT GTT CGT TCG GCT CGC GTG ATT TGG TCC CCT TCA ACC AGC TAT AAT CTC AGC-3′). miR-CVB3-2.2 was generated by first inserting TS of miR-1 and miR-216 (one copy of each) between VP2 and VP3 using a primer pair (forward: 5′- GGCTTACCAACCATGAATAC-3′, reverse: 5′- CTGGTGCCCTGCTAAACGTAAC-3′) and an oligo pair (fragment 1: 5′-GTT ACG TTT AGC AGG GCA CCA GGG CCT TAA AGA CAT ACA TAC TTC TTT ACA TTC CAA TAG TCA CAG TTG CCA GCT GAG ATT AGC AGA GTT TCA AGG CTT ACC AAC CAT GAA TAC-3′), followed by further insertion of TS of miR-133 and miR-375 (one copy of each) between VP3 and VP1 with a primer pair (forward: 5′-GGCCCAGTGGAAGACGCGATA-3′, reverse: 5′-CTGGAAAAAGTTTTGCTGCG-3′) and an oligo pair (fragment: 5′-CGC AGC AAA ACT TTT TCC AGG GTC CAC CAG TAC TTA GCT GGT TGA AGG GGA CCA AAA CTA GTC ACG CGA GCC GAA CGA ACA AAG CAC TAT TCC AGG GCC CAG TGG AAG ACG CGA TA-3′). The live viral stock was then made as previously described.^5^

### Animal studies

A/J mice (stock #000646) and immunocompromised non-obese diabetic-severe combined immune deficiency (NOD-SCID) mice (NOD.CB17-*Prkdc*^*scid*^/J, stock #001303) were purchased from the Jackson Laboratory. All mouse experiments were conducted at the Center for Heart Lung Innovation Animal Facility of the University of British Columbia in strict accordance with the recommendation in the Guide for the Care and Use of Laboratory Animals (Canadian Council on Animal Care, Ottawa, ON, Canada). The animal protocol related to this study was approved by the University Animal Care Committee (A18-0275).

To evaluate the safety of newly generated miR-CVB3s, male A/J mice at the age of ∼4–5 weeks were inoculated once intraperitoneally with PBS (sham), WT-CVB3, or various miR-CVB3s at 1 × 10^6^ PFU for up to 14 days (experimental endpoint). The animals were then monitored daily, and body weight was measured every 2 days. Mice were euthanized prior to the experimental endpoint per the approved animal protocol in the case of morbidity exceeding the humane criteria. Different cohorts of mice were treated as above, and mouse organs were harvested on day 4 for histological examination and immunostaining of viral capsid protein VP1.

To assess the safety and anti-tumor efficacy of miR-CVB3s, male NOD-SCID mice at the age of ∼6–8 weeks were injected subcutaneously with H526 cells (1 × 10^7^ cells) for the establishment of xenograft tumor in the right flank. Once the tumor volume reached a palpable size (∼100 mm^3^), mice were inoculated intraperitoneally with miR-CVB3-1.1, miR-CVB3-2.1, or miR-CVB3-2.2 at a dose of 1 × 10^6^ PFU. Mice treated with an equal volume of PBS were used as controls. miR-CVB3 injection was repeated weekly till the tumor growth stably suppressed. The animals were monitored daily, and body weight was examined twice every week. The tumor size was measured twice weekly until the experimental endpoint (150 days after viral injection) and tumor volume was calculated as (length × width^2^ × 0.52). If the tumor length exceeded 20 mm in diameter or mice presented severe symptoms linked to viral infection, the mice were euthanized prior to the experimental endpoint per the approved animal protocol.

### Reverse transcription-quantitative polymerase chain reaction (qRT-PCR)

The relative expression of miR-1, miR-133, miR-216, and miR-375 was determined by qRT-PCR as previously described.^6^^,^^17^ In brief, stem loop primers: miR-1 (5′-GTCGTATCCAGTGCAGGGTCCGAGGTATTCGCACTGGATACGACATACAT-3′), miR133 (5′-CTCAACTGGTGTCGTGGAGTCGGCAATTCAGTTGAGTAGCTGGT-3′), miR-216 (5′-GTC GTA TCC AGT GCA GGG TCC GAG GTA TTC GCA CTG GAT ACG ACT CAC AG-3′), miR-375 (5′- GTCGTATCCAGTGCAGGGTCCGAGGTATTCGCACTGGATACGACTCACGC-3′), and miR-93 (internal control, 5′-CTCAACGGTGTCGTGGAGTCGGCAATTCAGTTGAGCTACCTGC-3′) were used for RT with an iScript cDNA synthesis kit (#1708890, Bio-Rad), followed by qPCR analysis on a ViiA 7 real-time PCR system using respective primers: miR-1 (forward: 5′-TGG AAT GTA AAG AAG TAT GTA T-3′, reverse: 5′-GTG CAG GGT CCG AGG T-3′), miR-133 (forward: 5′- CAGGTTTGGTCCCCTTCAA-3′, reverse: 5′- TCAACTGGTGTCGTGGAGTC-3′), miR-216 (forward: 5′-AAC ACG TGT AAT CTC AGC TGG-3′, reverse: 5′-GTC GTA TCC AGT GCA GGG T-3′), miR-375 (forward: 5′- AACACGCTTTGTTCGTTCGG-3′, reverse: 5′- GTCGTATCCAGTGCAGGGT-3′), and miR-93 (forward: 5′-CGGCGGCAAAGTGCTGTTCGTG-3′, reverse: 5′-CTGGTGTCGTGGAGTCGGCAATTC-3′). Samples were run in triplicate, analyzed using the comparative CT (2^−ΔΔCT^) method and presented as relative quantitation of miR-1, miR-133, miR-216 and miR-375 to miR-93.

For viral RNA quantitation in mouse tissues, total RNA was extracted using the Monarch total RNA miniprep kit (#T2010, New England Biolabs) and aliquoted to 20 ng/μL. Viral RNA levels were measured by qRT-PCR using primers targeting CVB3 2A (forward: 5′-GCTTTGCAGACATCCGTGATC-3′, reverse: 5′-CAAGCTGTGTTCCACATAGTCCTTCA-3′). The genome copy number was then calculated based on the standard curve made using viral RNA acquired from *in vitro* transcription. Organs harvested from PBS-treated mice were used to measure the background and/or unspecific amplification.

### Stability examination in HL-1 cells

To test the genomic stability, the newly established miR-CVB3s were serially passaged in HL-1 cardiomyocytes (i.e., supernatant from infected cells was collected and transferred to new cells) for 20 rounds (n = 3 wells for each round). The cell supernatants were collected every five passages at 24 h post-infection for viral genome quantification by qRT-PCR with primer pairs as follows: miR-CVB3-1.1 (forward: 5′-CCCTTTGTTGGGTTTATACCACTT-3′, reverse: 5′-CCAGGAATCCCTTTGACGTCCA-3′), miR-CVB3-2.1 (forward: 5′-CCATATAGCTATTGGATTGGCCAT-3′, reverse: 5′-CGTTGATACTTGAGCTCCCAT-3′), miR-CVB3-2.2 site 1 (forward: 5′-GCCGAGTACAATGGGTTACG-3′, reverse: 5′-CTGGCAACTGTGACTATTGGAA-3′), miR-CVB3-2.2 site 2 (forward: 5′-GTTGAAGGGGACCAAAACTAGTC-3′, reverse: 5′-CTCCCTATAGCGGCTGTTATCG-3′), CVB3 2A (see section above), and CVB3 P1 (forward: 5′-GAAGGACACTCCTTTCATTTCGC-3′, reverse: 5′-CTCCCTATAGCGGCTGTTATCG-3′). For the relative quantification analysis of miR-TS in various miR-CVB3s, 2A of CVB3 was used as a reference control (Figure 2A). Absolute viral RNA level in supernatant was measured using CVB3 2A primers as described above.

### Histological examination and immunohistochemical staining

Potential tissue toxicity was evaluated by histological analysis following H&E staining. Immunohistochemical staining was performed using the primary monoclonal anti-CVB3 capsid protein VP1 antibody (1:1,200, Cox mAB 31A2, Mediagnost, Germany) as described previously.^6^ VP1 staining was quantified by ImageJ (version 1.52p) and presented as relative optical density normalized to sham infection as previously reported.^23^

### Cell viability assay

Cell viability was evaluated using alamarBlue according to the manufacture’s protocol (#BUF012A, Bio-Rad Laboratories). Briefly, the alamarBlue solution was added to the 48-well plate to a final concentration of 10%. After incubation at 37°C for 4 h, the absorbance was measured at 570 nm and 600 nm on a microplate reader. Percentage survival of CVB3-infected cells is expressed relative to that of sham control, which is arbitrarily set as 100% survival.

### Blood chemistry test

Blood biochemistry analysis for lipase (unit/L) levels was performed using Siemens Advia 1800 analyzer at the clinical laboratory of St. Paul’s Hospital (Vancouver, Canada).

### Statistical analysis

All results are expressed as mean ± standard deviation (SD). Statistical analysis was conducted using one-way ANOVA or unpaired Student’s t test with GraphPad Prism 9. The log rank test was used to compare the survival rate between the groups throughout the whole monitoring period. A value of p < 0.05 is considered statistically significant.

## Data availability statement

The data that support the findings of this study are available on request from the corresponding author.

## Supplemental information

Document S1. Figures S1 and S2

Document S2. Article plus supplemental information
