# Supplementary material for: Enhanced genomic stability of new miRNA-regulated oncolytic coxsackievirus B3
Source: Mol Ther Oncolytics. 2022 Oct 8;27:89–99. doi: 10.1016/j.omto.2022.10.003 (PMC9593271; doi:10.1016/j.omto.2022.10.003)
Supplement: Document S1. Figures S1 and S2 [file mmc1.pdf]

OMTO, Volume *27*

Supplemental information

Enhanced genomic stability of new miRNA-regulated oncolytic coxsackievirus B3

Huitao Liu, Amirhossein Bahreyni, Yasir Mohamud, Yuan Chao Xue, William W.G. Jia, and Honglin Luo

miR-CVB3-2.1

```
                         miR-1-TS                     miR-216-TS                    miR-133-TS                   miR-375-TS
Original atccggtgATACATACTTCTTTACATTCCATCACAGTTGCCAGCTGAGATTATAGCTGGTTGAAGGGGACCAAATCACGCGAGCC GAA C GAA CAAAataca
      #1 atccggtgATACATACTTCTTTACATTCCATCACAGTTGCCAGCTGAGATTATAGCTGGTTGAAGGGGACCAAATCACGCGAGCCCGAA CCGAA CCCAat
      #2 atccggtgATACATACTTCTTTACATTCCATCACAGTTGCCAGCTGAGATTATAGCTGGTTGAAGGGGACCAAATCACGCGAGCC GAAAC GAA -A-----cc
      #3 atccggtgATACATACTTCTTTACATTCCATCACAGTTGCCAGCTGAGATTATAGCTGGT-GAAGGG-ACCAAATCACGCGAGCCCGAAACCGAAACCAA
```

miR-CVB3-2.2

```
                         miR-1-TS                    miR-216-TS                                  VP3
Original ttqaagacATACATACTTCTTTACATTCCAatagTCACAGTTGCCAGCTGAGATTAgcagagtttcaaggcttaccaaccatgaatactccggggagctg
      #1 ttaaagacATACATACTTCTTTACATTCCAatagTCACAGTTGCCAGCTGAGATCAgcagagtttcaaggcttaccaaccatgaatactccggggagctg
      #2 ttaaagacATACATACTTCTTTACATTCCAatagTCACAGTTGCCAGCTGAGATTAgcagagtttcaaggcttaccaaccatgaatactccggggagctg
      #3 ttaaagacATACATACTTCTTTACATTCCAatagTCACAGCTGCCAGCTGAGATTAgcagagtttcaaggcttaccaaccatgaatactccggggagctg

                          VP3                                    miR-133-TS                 miR-375-TS
Original ttcatttcgcagcaaaactttttccagggtccaccagtactTAGCTGGTTGAAGGGGACCAAAactagTCACGCGAGCCGAACGAACAAAgcactatt
      #1 ttcatttcgcagcaaaactttttccagggtccaccagtactTAGCTGGTTGAAGGGGACCAAAactagTCACGCGAGCCGAACGAACAAAgcactatt
      #2 ttcatttcgcagcaaaactttttccagggtccaccagtactTAGCTGGTTGAAGGGGACCAAAactagTCACGCGAGCCGAACGAACAAAgcactat-
      #3 ttcatttcgcagcaaaactttttccagggtccaccagtactTAGCTGGTTGAAGGGGACCAAAactagTCACGCGAGCCGAACGAACAAAgcactatt
```

Figure S1. Three hearts (i.e., #1, #2, and #3) harvested from A/J mice from Figure 4, which were treated with miR-CVB3-2.1 or miR-CVB3-2.2 for 14 days, were subjected to RNA extraction and Sanger sequencing. The mutation is highlighted in red. “-” denotes missing nucleotide.

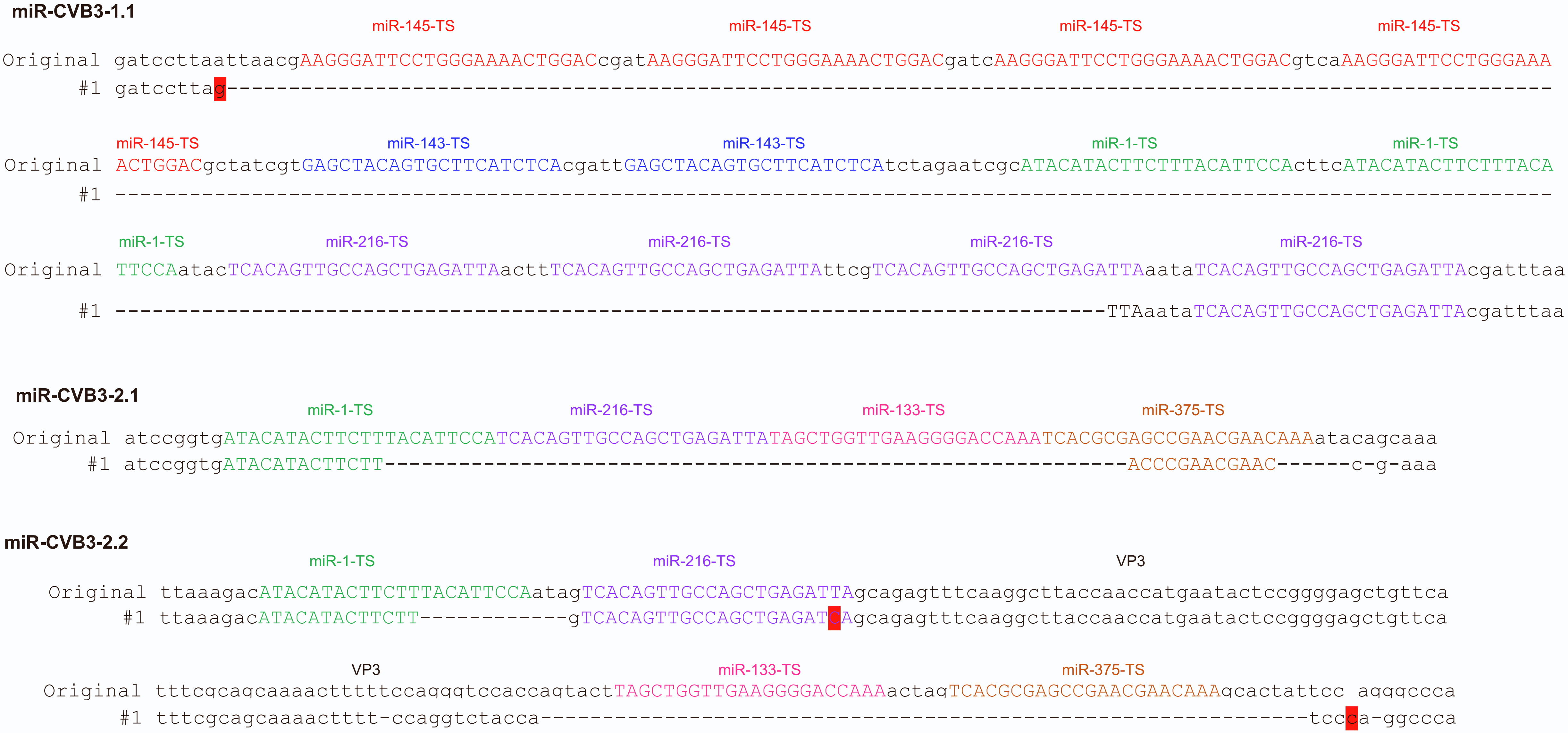

Figure S2. Total RNA was extracted from the hearts of miR-CVB3-treated NOD-SCID mice from Figure 5, which died prior to the experimental endpoint of day 150 (i.e., miR-CVB3-1.1 on day 47, miR-CVB3-2.1 on day 72 and miR-CVB3-2.2 on day 112) for examination of the integrity of miRNA target sequence by Sanger sequencing. The mutation is highlighted in red. “-” denotes missing nucleotide.
